# Public perceptions of trophy hunting are pragmatic, not dogmatic

**DOI:** 10.1098/rspb.2023.1638

**Published:** 2024-02-14

**Authors:** Darragh Hare, Amy J. Dickman, Paul J. Johnson, Betty J. Rono, Yolanda Mutinhima, Chris Sutherland, Salum Kulunge, Lovemore Sibanda, Lessah Mandoloma, David Kimaili

**Affiliations:** ^1^ Department of Biology, University of Oxford, Oxford, UK; ^2^ Wildlife Conservation Research Unit, The Recanati-Kaplan Centre, University of Oxford, Oxford, UK; ^3^ Department of Natural Resources and the Environment, Cornell University, Ithaca, NY, USA; ^4^ Department of Zoology and Entomology, Rhodes University, Grahamstown, South Africa; ^5^ Department of Wildlife Ecology and Conservation, Chinhoyi University of Technology, Chinhoyi, Zimbabwe; ^6^ Centre for Research into Ecological and Environmental Modelling, School of Mathematics and Statistics, St Andrews University, St Andrews, UK; ^7^ Department of Wildlife Management, Sokoine University of Agriculture, Morogoro, Tanzania; ^8^ Tanzania Wildlife Management Authority, Morogoro, Tanzania; ^9^ Cheetah Conservation Project Zimbabwe, Dete, Zimbabwe; ^10^ Department of Sociology and Anthropology, South Eastern Kenya University, Kitui, Kenya

**Keywords:** conservation conflicts, politics, social acceptability, sustainable use, wildlife conservation

## Abstract

Fierce international debates rage over whether trophy hunting is socially acceptable, especially when people from the Global North hunt well-known animals in sub-Saharan Africa. We used an online vignette experiment to investigate public perceptions of the acceptability of trophy hunting in sub-Saharan Africa among people who live in urban areas of the USA, UK and South Africa. Acceptability depended on specific attributes of different hunts as well as participants' characteristics. Zebra hunts were more acceptable than elephant hunts, hunts that would provide meat to local people were more acceptable than hunts in which meat would be left for wildlife, and hunts in which revenues would support wildlife conservation were more acceptable than hunts in which revenues would support either economic development or hunting enterprises. Acceptability was generally lower among participants from the UK and those who more strongly identified as an animal protectionist, but higher among participants with more formal education, who more strongly identified as a hunter, or who would more strongly prioritize people over wild animals. Overall, acceptability was higher when hunts would produce tangible benefits for local people, suggesting that members of three urban publics adopt more pragmatic positions than are typically evident in polarized international debates.

## 1. Introduction

What if the answer to the question *Is trophy hunting acceptable*? was not *yes* or *no*, but *it depends*?

'Trophy hunting' refers to a category of legal hunting in which people pay to hunt animals and keep body parts as mementos of the experience [1,2]. Although a wide range of species are hunted for trophies, fierce international public debates rage over whether any form of trophy hunting is socially acceptable [3–9], and it appears especially controversial when people from the Global North hunt well-known animals such as elephants, giraffes, leopards, lions and zebras in sub-Saharan Africa and keep trophies [7,10–14].

Much of the public debate surrounding trophy hunting in sub-Saharan Africa occurs on social and in traditional media [5,15–17]. Many of the most outspoken contributors are based outside of sub-Saharan Africa, including the UK and USA. These include animal welfare and protection advocates who argue trophy hunting is cruel, threatens wildlife populations, exacerbates human–wildlife conflict, and provides little economic benefit to local communities, as well as hunting and conservation advocates who argue trophy hunting can generate revenues which support wildlife conservation and economic development, and therefore provide local incentives for local communities to maintain habitat and tolerate dangerous wild animals.

Social media exchanges tend to be polarized and acrimonious, reflecting these divergent positions [5,16,17]. Traditional media coverage tends to present a simplistic narrative that trophy hunting is categorically immoral and detrimental to wildlife conservation [18]. For example, UK newspapers frequently assert that trophy hunters kill iconic animals and contribute to biodiversity decline, but seldom mention evidence that well-regulated, community-led trophy hunting can generate sustainable direct or indirect benefits for wildlife populations and for people who live in rural areas where livelihood options can be limited [13]. Documented benefits from well-regulated trophy hunting include employment in hunting and related industries, meat from hunted animals, and revenues that can defray substantial costs associated with wildlife conservation and support economic development, for example through investments in infrastructure, healthcare and education [14,19–28].

Some members of the international conservation community argue that if movements to restrict or ban trophy hunting succeed, they will generate unintended negative consequences for people, wild animal welfare and wildlife conservation [3,18,22,29–31]. Other members of the international conservation community point to examples of poorly regulated hunting systems that have negatively affected wildlife populations or failed to deliver local economic benefits [32,33], and some assert that trophy hunting is unacceptable on moral grounds that outweigh consequences for people or wildlife populations [4,34].

Decisions in rural areas of sub-Saharan Africa where trophy hunting does or could occur can be affected by perceptions of the acceptability of trophy hunting among external publics (i.e. those who live outside of sub-Saharan Africa or in urban areas of sub-Saharan Africa). Although governments in sub-Saharan Africa as well as private and community landholders in some countries have legal rights to manage wildlife, external organizations can influence their wildlife management decisions, including whether or not to allow trophy hunting on land under their jurisdiction [35]. For example, financial, political or technical support for conservation and economic development programmes may carry stipulations that hunting must or must not be allowed for certain species or in particular places. In the case of non-governmental organizations, such stipulations may reflect the values and preferences of their staff, trustees, donors or members. Similarly, support from governmental organizations may be contingent on the values and preferences of external political administrations, or how those administrations perceive the values and preferences of the people they represent.

Relatedly, legislation preventing trophy imports to North America or Europe—the two largest markets for trophy hunting in sub-Saharan Africa—could, even if the number of animals hunted for trophies is small, influence decisions on whether to maintain large areas of land in sub-Saharan Africa for wildlife habitat or use it for competing purposes such as crop or livestock production [25,29,30]. Alternative conservation-orientated land uses, such as photographic tourism, are chronically underfunded, vulnerable to economic shocks, and not viable in many areas where hunting does or could take place [8,21,28,36]. Those alternatives are therefore unlikely to compete at the necessary scale with land use options that are more profitable and more predictable but less conducive to wildlife conservation, such as agriculture.

In all of these cases, there is potential for external people's values and preferences to conflict with, and possibly override, the values and preferences of local people who bear the costs of sharing landscapes with wildlife. Although research into the diversity of African views on trophy hunting is limited [5,7,14], people living in several rural areas of sub-Saharan Africa perceive community-led trophy hunting to be an acceptable component of wildlife management and an important source of economic benefits [24,29,37–39]. Nevertheless, local decisions to allow trophy hunting can meet staunch external opposition, illustrating the international nature of debates and highlighting ethical concerns about external interests constraining self-determination in rural areas of sub-Saharan Africa [5,7,35,40–42].

A detailed understanding of how acceptable or unacceptable members of external publics perceive trophy hunting to be could help inform decisions over its role in wildlife management and economic development. These include decisions in African countries on whether to continue, reform or establish hunting operations as well as decisions on potential trophy import restrictions in North America and Europe. Such a detailed understanding does not currently exist because of a lack of research directly investigating the contours of public opinion on the topic. Among people living in the USA and the UK, trophy hunting appears to be less acceptable than other forms of hunting [43–47]. However, there is some evidence that people perceive trophy hunting to be more acceptable if they learn about potential positive outcomes associated with it. For example, while members of the UK public appear generally unsupportive of trophy hunting in sub-Saharan Africa, that opposition is less pronounced when participants are informed that trophy hunting can provide benefits to wildlife conservation and rural African people [48].

Similarly, although a majority of adults responding to a poll in the USA disapproved of trophy hunting, more than one-third of those who disapproved said their opinion would change if revenues from hunting improved conservation outcomes [46]. Moreover, hunters from North America and Europe appear sensitive to how hunting revenues are used, reporting willingness to pay a higher price to hunt in landscapes with abundant wildlife populations and when revenues provide greater benefits to local people and conservation [49,50]. These patterns suggest that members of external publics perceive some forms of trophy hunting to be more or less acceptable than others [9,51]. However, exactly which forms of trophy hunting are perceived to be more or less acceptable, the extent to which perceptions of acceptability are consistent across different external publics, and whether perceptions vary with demographic profiles, social identities or orientations towards people and wild animals [6,16] are currently unknown.

Here we report results from an online experiment investigating perceptions of the acceptability of trophy hunting well-known animals in sub-Saharan Africa among people who live in urban areas of the USA, UK and South Africa (SA). Our objective is to fill knowledge gaps and bring greater clarity to international debates over the acceptability of trophy hunting as a component of wildlife management [6,52–54] by constructing an evidence-based understanding of how members of three external publics think about the acceptability of trophy hunting in sub-Saharan Africa. We test the following research hypotheses (our *a priori* predictions about the direction of effects are in the electronic supplementary material, S1):

H_1_. acceptability of trophy hunting will depend on:
  H_1a_. which animal would be hunted;
  H_1b_. how meat from the hunt would be used;
  H_1c_. how revenue from the hunt would be used;
  H_1d_. which country participants come from;
H_2_. effects of which animal would be hunted, how meat would be used, and how revenue would be used will differ for participants from the USA, UK and SA; and
H_3_. participants' characteristics, for example, demographic profiles, social identities and beliefs about conservation priorities.

## 2. Methods

### (a) Participants and sampling

We hired Qualtrics (www.qualtrics.com) to recruit participants living in urban areas of the USA, UK and SA. To increase external validity [55], our samples for each country approximated national population estimates for gender and ethnicity [56–58] and were close to evenly split across three age groups: 18–35, 36–55 and 56 or older (electronic supplementary material, S2).

We chose to study acceptability among people living in urban areas because these tend to be population centres where political and economic power are concentrated. We sampled people living in the USA, UK and SA to encompass some diversity in external urban perspectives, specifically related to contrasting hunting cultures and traditions. Only a small proportion of the UK public hunt, although hunting (including trophy hunting) takes place in many rural areas, especially of red deer (*Cervus elaphus*) in Scotland [45,59]. There is a strong public hunting culture in the USA, which includes trophy hunting [47,60], and the majority of international trophy hunters in sub-Saharan Africa travel from the USA [12,26]. There is also a strong public hunting culture in SA, and income from trophy hunting contributes substantially to its wildlife economy [27,28].

### (b) Experimental design

We used experimental vignettes [61] to evaluate whether perceptions of the acceptability of trophy hunting in sub-Saharan Africa depend on three factors: the animal that would be hunted (two levels: elephant or zebra), how meat from the hunt would be used (two levels: left for wildlife or provided to local people), and how revenue from the hunt would be used (three levels: support conservation in the area, support economic development in the area, or support hunting enterprises in the area). A full-factorial (2 × 2 × 3) design comprising all possible combinations of each factor level produced 12 experimental conditions.

**Figure 1.**
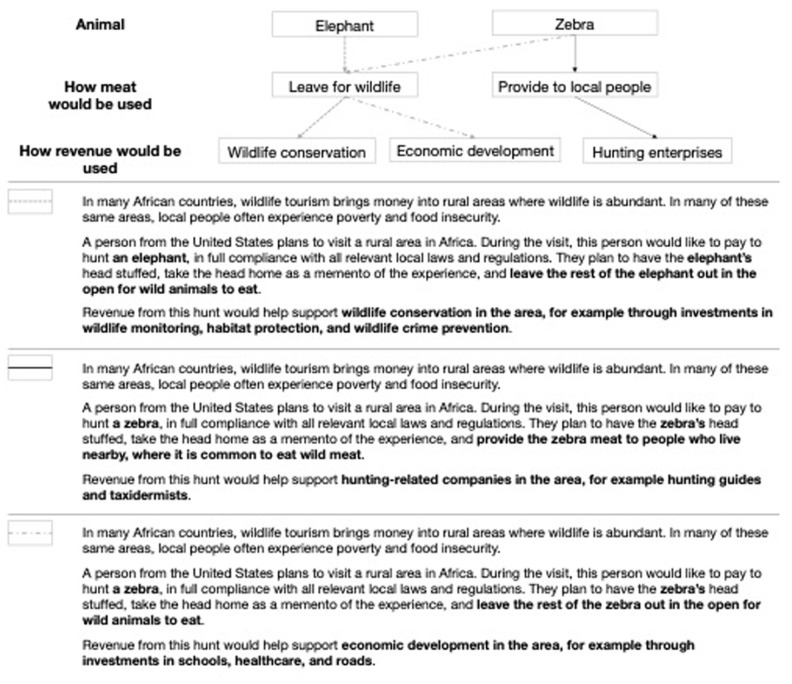
Experimental design and example vignettes. A full-factorial (2 × 2 × 3) design comprising all possible combinations of each factor and level produced 12 experimental conditions. Examples show unique vignettes corresponding to three conditions. All vignettes were identical except for the language we manipulated, representing different levels of each factor.

For each experimental condition, we constructed a unique vignette, i.e. a passage describing a hypothetical scenario in which a tourist hunts in sub-Saharan Africa and keeps the animal's head as a memento. All vignettes were identical except for the language we manipulated, corresponding with different levels of our three experimental factors: the animal that would be hunted, how the meat would be used, and how the revenue would be used (figure 1). Each vignette began with an identical statement that wildlife tourism (inclusive of all types of tourism, not just hunting) brings income to rural areas in sub-Saharan Africa where wildlife is abundant but local people often experience poverty and food insecurity, then described a discrete scenario in which a tourist from the USA would like to hunt in an unspecified African country (figure 1; electronic supplementary material, S3).

We selected two well-known African herbivores, elephants and zebras, to ensure that participants, especially those from the UK and USA, would be familiar enough with the animals to meaningfully interpret the scenarios described in vignettes. Both elephants and zebras are perceived to be among the most charismatic wild animals internationally, with elephants perceived as more charismatic than zebras [62]. The International Union for the Conservation of Nature (IUCN) lists African savannah elephants (*Loxodonta africana*) as endangered and African forest elephants (*Loxodonta cyclotis*) as critically endangered. However, the IUCN lists only one of the three extant zebra species, Grevy's zebra (*Equus grevyi*) as endangered [63].

In our vignettes, we chose not to use the term ‘trophy hunting', which may be vulnerable to inaccurate interpretations (e.g. possible conflation with canned hunting or poaching [64]), evoke inaccurate stereotypes [18], or obscure important differences between types of hunts [9]. Instead, we described specific attributes of hunts using plain language and terms intended to be as value-neutral as possible. We pre-tested vignettes among researchers not involved in the study (*n* = 9) for understandability, value-neutrality, and relevance to international debates about trophy hunting, and refined vignettes according to their feedback.

### (c) Procedure

Qualtrics directed participants to our online instrument where we randomly assigned one vignette to each participant. After reading the vignette, participants indicated their perception of how acceptable it would be for the specific hunt described in that vignette to take place, using a 7-point bipolar Likert-type scale ranging from ‘very unacceptable' to ‘very acceptable', with a central point of ‘neither acceptable nor unacceptable'. We also made the additional response option of ‘I don't know' available to participants to distinguish those who perceived the hunt described in vignettes to be neutral in terms of acceptability (neither acceptable nor unacceptable) from those who did not know how acceptable they perceived the hunt to be. Participants then reported their demographic characteristics (age, ethnicity, gender, level of formal education, and whether they grew up in a rural or urban location), social identities (the extent to which they consider themselves to be a hunter, conservationist, animal protection advocate and human rights advocate), and orientations towards people and animals (how they would prioritize the interests of people versus the interests of wild animals, and how they would prioritize the interests of individual wild animals versus groups of wild animals). The full instrument is available in the electronic supplementary material, S4.

We conducted a soft launch (*n* = 383; 202 from the USA, 116 from the UK and 65 from SA) to test instrument functionality, and made no subsequent changes to the instrument before continuing data collection until we met our targets for gender, ethnicity and age for all countries (*n* = 1225; 418 from the USA, 401 from the UK and 406 from SA). The median time for completing all sections of the instrument during the soft launch was 194 s. We excluded responses from any participants who took an unacceptably short (less than 97 s, half the median) or unacceptably long (greater than 776 s, four times the median) time to complete all sections of the instrument [55]. All participants provided informed consent before answering any items.

We removed 25 responses from participants who answered ‘I don't know' when asked how acceptable it would be for the hunt described in the vignette they read to take place, retaining only responses on the scale from very unacceptable to very acceptable. We also removed responses from eight participants identifying as transgender, non-binary, other gender, or preferring not to disclose their gender identity as this was too few to include as a factor in our model. Our final dataset therefore contained 1192 responses (404 from the USA, 388 from the UK and 400 from SA) across 12 experimental conditions (median number per condition: 99; range: 89–118).

### (d) Data analysis

We used ordinal logistic regression to quantify associations between respondents' perceptions of the acceptability of trophy hunting (i.e. their indications of how acceptable it would be for the specific hunt described in the vignette they read to take place) and our three experimental factors (the animal that would be hunted, how the meat would be used, and how the revenue would be used), accounting for participants' country of residence, age, ethnicity, gender, formal education, whether they grew up in a rural or urban location, the extent to which they identify as a hunter, conservationist, animal protection advocate and human rights advocate, whether they would prioritize people or wild animals when their interests clash, and whether they would prioritize individual wild animals versus groups of wild animals when their interests clash.

To test our hypotheses, we fitted a global model containing all possible two-way interactions among participants' country of residence and our three experimental factors, and main effects of all other predictors (electronic supplementary material, S5). We used corrected Akaike information criterion (AIC_c_) to compare the global model and all possible models nested within it. We identified a top-supported model, i.e. the model with the lowest AIC_c_ (electronic supplementary material, S6), and computed AIC_c_ weights (AIC_w_) for all models with AIC_c_ values within 2 of the top-supported model after removing uninformative or redundant parameters [65–67] (electronic supplementary material, S7). We used Tukey tests, adjusted for unequal numbers of observations among groups, to assess differences between levels of each categorical predictor variable with more than two levels in the top-supported model. We calculated relative acceptability of hunts described in each of the 12 hypothetical scenarios while accounting for all predictor variables in our top-supported model (i.e. marginal predictions for all 12 combinations of our three experimental factors).

We report results from the top-supported model and visualize estimates and predictions with 85% confidence intervals, consistent with using AIC_c_ for model selection [67], as well as conventional 95% confidence intervals. We analysed data in R, version 4.3.0 [68], using: the ordinal package [69] to fit models; the MuMIn package [70] for model selection and comparison; the emmeans package [71] for post hoc tests and to calculate marginal predictions; the Likert package [72] to visualize raw data; and the ggplot2 [73] package to visualize parameter estimates and predictions from the top-supported model. Datasets and code for data analyses are available at Dryad [74].

## 3. Results

**Figure 2.**
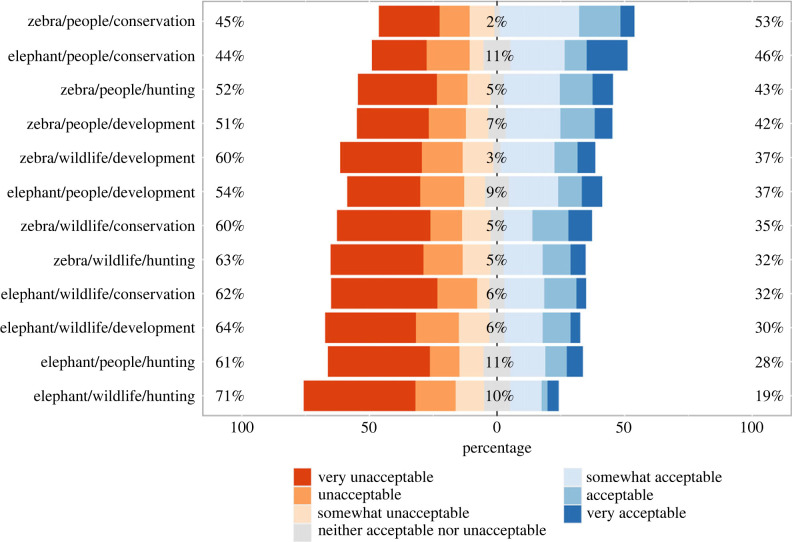
Acceptability of trophy hunting in 12 experimental scenarios. Each bar represents one scenario describing a hunt involving either an elephant or a zebra, in which the meat would be provided to people living in the area or left for wildlife, and the revenue would help support wildlife conservation, economic development or hunting enterprises. Colours show distribution of responses. Percentages show combined proportions of participants who indicated that the hunt would be very unacceptable, unacceptable, or somewhat unacceptable (left), neither acceptable nor unacceptable (middle), or somewhat acceptable, acceptable or very acceptable (right), after excluding ‘I don't know' responses.

Acceptability of trophy hunting varied across our experimental scenarios (figure 2). The least acceptable scenario overall was hunting an elephant with the meat left for wildlife and revenue supporting hunting enterprises (70.7% of participants perceiving it to be somewhat unacceptable, unacceptable, or very unacceptable). The same scenario had the highest proportion of participants perceiving it to be very unacceptable (43.8%). The most acceptable scenario overall was hunting a zebra with the meat going to local people and revenue supporting conservation (52.8% of participants perceiving it to be somewhat acceptable, acceptable, or very acceptable). The scenario with the highest proportion of participants perceiving it to be very acceptable (16.0%) was hunting an elephant with the meat going to local people and revenue supporting conservation.

Our top-supported model (AIC_w_ = 0.53, electronic supplementary material S6 and S7) contained main effects of all three experimental factors, along with participants’ highest level of formal education, the degree to which they identify as a hunter, the degree to which they identify as an animal protectionist, and their beliefs about whether to prioritize people or wild animals when their interests clash. All other variables, including all interaction terms, dropped out during the model selection process.

All else equal, participants were more likely to perceive zebra hunts as more acceptable than elephant hunts (difference (s.e.) in log odds ratio = 0.23 (0.11)), hunts in which the meat would be provided to local people as more acceptable than hunts in which the meat would be left for wildlife (difference (s.e.) in log odds ratio = 0.49 (0.11)) and hunts in which the revenue would help support wildlife conservation as more acceptable than hunts in which the revenue would help support economic development (Tukey test, difference (s.e.) in log odds ratio = 0.19 (0.13)) or hunting enterprises (Tukey test, difference (s.e.) in log odds ratio = 0.43 (0.13)). Participants were more likely to perceive hunts in which the revenue would help support economic development as more acceptable than hunts in which the revenue would help support hunting enterprises (difference (s.e.) in log odds ratio = 0.23 (0.13)).

**Figure 3.**
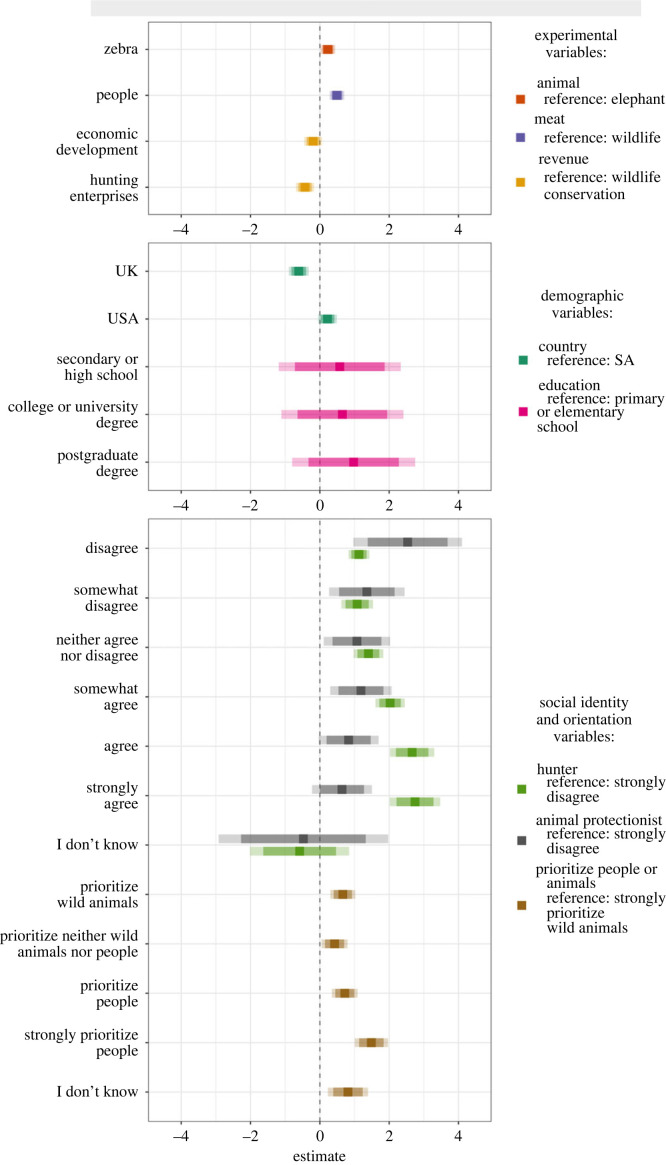
Associations between acceptability of trophy hunting and predictor variables in top-supported model. Squares show parameter estimates (log odds ratios) for each level of all experimental, demographic, and social identity and orientation predictor variables in the top-supported model relative to the reference category for that variable. Darker error bars show 85% confidence intervals and lighter error bars show 95% confidence intervals. Positive estimates indicate increased acceptability relative to the reference category, negative estimates indicate decreased acceptability relative to the reference category and estimates of zero indicate no difference in acceptability relative to the reference category.

**Figure 4.**
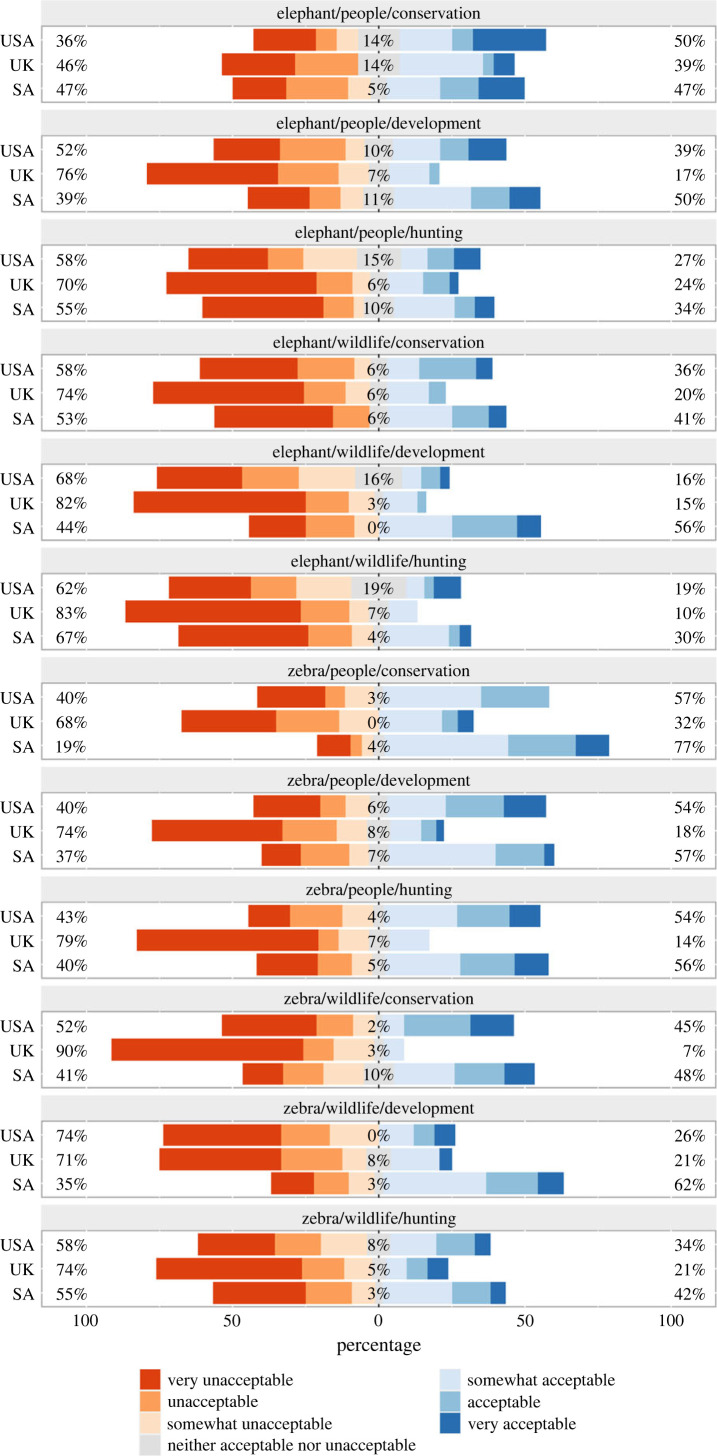
Acceptability of trophy hunting in 12 experimental scenarios, grouped by responses from the USA, UK and South Africa (SA). Each panel represents one scenario describing a hunt involving either an elephant or a zebra, in which the meat would be provided to people living in the area, and the revenue would help support wildlife conservation, economic development or hunting enterprises. Bars are grouped by participants from each country, and colours show distribution of responses. Percentages show combined proportions of participants from each country who indicated that the hunt would be very unacceptable, unacceptable, or somewhat unacceptable (left), neither acceptable nor unacceptable (middle), or somewhat acceptable, acceptable or very acceptable (right), after excluding ‘I don't know' responses.

Participants' country of residence helped explain variation in acceptability of trophy hunting (figures 3 and 4). All else equal, participants from the USA were more likely to perceive trophy hunting as more acceptable than participants from SA (difference (s.e.) in log odds ratio = 0.22 (0.14)) and the UK (difference (s.e.) in log odds ratio = 0.83 (0.14)). Participants from SA were more likely to perceive trophy hunting as more acceptable than participants from the UK (difference (s.e.) in log odds ratio = 0.61 (0.15)).

Participants' sociodemographic characteristics, social identities and beliefs about whether to prioritize the interests of people or animals also helped explain variation in acceptability of trophy hunting (figure 3). Acceptability generally increased with more formal education and identifying more strongly as a hunter. Acceptability generally decreased with more strongly identifying as an animal protectionist. Acceptability was higher among participants who would strongly prioritize the interests of people over the interests of wild animals compared to those who reported any other belief about whether to prioritize the interests of people or wild animals when their interests clash.

## 4. Discussion

We used experimental vignettes to evaluate how people living in urban areas of the USA, UK and SA perceive the acceptability of trophy hunting across 12 hypothetical scenarios, each describing a different hunt. We found that perceptions of the acceptability of trophy hunting were not dogmatic but depended on specific attributes of the hunt (which animal would be hunted, how the meat would be used, and how the revenue would be used), as well as on participants' country of residence, degree of formal education, social identities and orientation towards people and wild animals. We found support for five out of our six research hypotheses, largely in the direction we predicted (electronic supplementary material, S1). The exception was that H_2_ was not supported: the effects of which animal would be hunted, how meat would be used, and how revenue would be used did not differ for participants from the UK, USA and SA (interactions between experimental factors and respondents' country of residence did not appear in our top-supported model).

Nevertheless, participants' country of residence did help explain differences in acceptability of trophy hunting. Overall, participants from the UK perceived the hunts described in our vignettes to be generally less acceptable than participants from the USA and SA. This result is clearly visible in figure 4, where for most scenarios the distribution of responses is left-shifted and the proportion of participants indicating ‘strongly disagree’ is consistently greater for UK participants (electronic supplementary material, S8). This difference might be at least partially attributable to differences in public hunting cultures. Hunting is generally less prevalent in the UK compared to the USA or SA, a contrast also reflected in the proportions of participants from each country identifying as hunters (electronic supplementary material, S2). Therefore, if people in the UK have less direct or indirect experience of hunting compared to people in the USA or SA, i.e. if there is less ‘social habitat’ for hunting [75] in the UK, they might perceive all forms of hunting to be less acceptable. While research into the contours of international disagreements over the acceptability of trophy hunting is currently limited [14], in this study, we found evidence for different baseline acceptability between countries but no evidence that the effects of any of our experimental factors varied between respondents from the USA, UK and SA. This reveals an intriguing consistency in how members of three different external publics evaluate the acceptability of trophy hunting under the hypothetical scenarios we provided.

**Figure 5.**
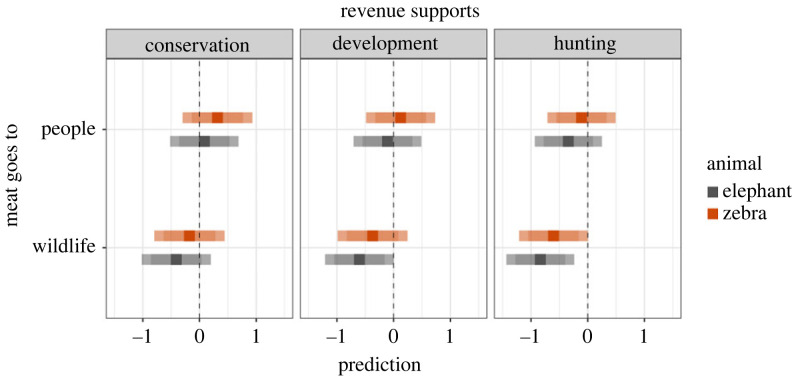
Predicted relative acceptability of hunting across 12 experimental scenarios. Squares show model-derived predictions for each combination of experimental factors (which animal would be hunted, how meat from the hunt would be used, and how revenue from the hunt would be used), accounting for effects of all other variables in our top-supported model (i.e. marginal predictions). Darker error bars show 85% confidence intervals and lighter error bars show 95% confidence intervals. Larger values indicate greater probability that, all else equal, a hunt would be perceived as more acceptable. All else equal, hunts involving zebras would be more acceptable than hunts involving elephants; hunts in which the meat would be provided to people are more acceptable than hunts in which the meat would be left for wildlife; and hunts in which the revenue would support conservation are more acceptable than hunts in which the revenue would support either economic development or hunting enterprises.

Participants generally perceived hunts involving zebras to be more acceptable than hunts involving elephants (figures 3 and 5). However, the effect of which animal would be hunted was not straightforward, with the four least acceptable scenarios overall involving elephant hunts but also the second most acceptable scenario overall involving an elephant hunt (figure 2). This particular scenario illustrates how multiple aspects of a hunt contribute to perceptions acceptability: the combined positive effects of meat being provided to local people and revenues helping support wildlife conservation offset the negative effect of hunting an elephant. Even when accounting for participants' demographic characteristics, social identities, and orientations towards people and wild animals, some hunts were perceived as more or less acceptable than others (figure 5).

Overall lower acceptability of hunting elephants compared to zebras could be interpreted as evidence that it is less acceptable to hunt species of greater conservation concern, with elephants generally experiencing higher threat than zebras [63]. Alternatively, this result could be interpreted as evidence that it is less acceptable to hunt more charismatic animals. Members of multiple publics, including the USA, UK and SA, tend to consider elephants to be more charismatic than zebras [62,76]. UK newspaper coverage and non-governmental organization reports critical of trophy hunting also tend to feature images of charismatic animals such as large carnivores and herbivores [10,12,13], reinforcing the possibility that some people may perceive hunting more charismatic animals to be less acceptable than hunting less charismatic animals.

In this study, we did not investigate directly whether perceived acceptability of trophy hunting depends on hunted animals' charisma, conservation status (e.g. endangered versus least concern), or other possibilities such as their feeding ecology (e.g. carnivore versus herbivore) or cultural value (e.g. the types of animals people venerate, vilify or eat in different societies [77]). We chose not to include a carnivore so that we could evaluate differences in acceptability of hunting different animals without introducing a potentially confounding effect of differences in feeding ecology, and so that we could test whether acceptability depends on whether meat from a hunt would be provided to local people or left for wild animals to eat. If charisma does predict acceptability of hunting, we would expect acceptability of hunting charismatic large carnivores such as lions (*Panthera leo*) among external publics to be roughly equivalent to acceptability of hunting elephants due to documented similarities in perceived charisma [62,76].

Our finding that hunting was more acceptable when meat would be provided to local people rather than left for wild animals (figures 3 and 5) is consistent with studies showing more favourable attitudes towards hunting for meat consumption than hunting for sport, pleasure or trophies [43,45,47], although these motivations are not mutually exclusive. The approach we employed challenges misleading dichotomies between hunting for trophies versus hunting for food. By describing scenarios in which hunts would produce both meat and trophies, we were able to disentangle the effects of these variables on perceptions of acceptability, and found that participants were sensitive to socio-economic aspects of hunting, such as how meat from hunts can contribute to food security in rural areas of sub-Saharan Africa [21,28]. In our hypothetical scenarios, we separated hunts in which meat would be left for wild animals or provided to local people, as well as hunts in which revenues would help support wildlife conservation, economic development or hunting enterprises. However, in reality, meat and revenue from any given hunt can be used for multiple purposes simultaneously [21]. Our finding that hunting was less acceptable when revenue helped support hunting enterprises than when it helped support wildlife conservation or economic development is consistent with a 2021 poll in the UK, which found that public support for a trophy hunting import ban decreased if the ban was likely to negatively affect marginalized rural people or wildlife conservation in Africa [46,48].

Taken together, our findings about the effects of how meat and revenue would be used indicate that outcomes of specific hunts matter to members of external publics when they evaluate the acceptability of trophy hunting [9,54,78]. Although some hunts described in our vignettes were on balance perceived towards the unacceptable end of our scale, none were perceived as categorically unacceptable (figures 2 and 4). Acceptability was generally higher for hunts that would produce tangible benefits for local people and when revenues would help support public service provision via wildlife conservation or economic development rather than help support private hunting enterprises (figures 3 and 5). These findings suggest members of external urban publics adopt more pragmatic stances than are typically evident in media coverage and social media exchanges that leave little room for context and nuance [13,16,17]. Furthermore, generally higher acceptability when hunts provide tangible local benefits reveals similarities in perceptions among participants in our study and people living in rural areas of sub-Saharan Africa who consider hunting to be an economically valuable and acceptable component of well-regulated community-led wildlife management systems [24,29,37–39].

We chose to describe specific attributes of different hunts rather than use the potentially misleading or provocative term ‘trophy hunting' in our vignettes, and found that differences between hunts are reflected in participants' perceptions of acceptability. Our results therefore underscore the importance of recognizing that trophy hunting is not a single activity, but a broad category that contains a range of activities that vary in terms of animals hunted, hunter motivations, effectiveness of regulation and governance structures, ecological impacts and economic impacts [9]. This finding is especially relevant as governments in North America and Europe continue to enact or consider legislation prohibiting or restricting trophy imports, with potentially serious ecological and economic ramifications for many rural areas of sub-Saharan Africa [25]. Some campaigns in favour of blanket bans of all trophy imports claim overwhelming public support among members of external publics, derived from opinion polls that do not differentiate between different types of trophy hunting [48,79]. Our finding that judgements of acceptability or unacceptability are sensitive to how revenues and meat would be used—with the most acceptable hunts contributing to conservation, economic development, and nutrition—suggests that ‘smart bans' on trophy imports, not blanket bans, would better reflect nuances in public opinion. Smart bans would allow imports from hunts that can clearly demonstrate local ecological and socio-economic benefits, and could therefore help drive positive reform within the hunting industry by incentivizing good practice [25].

Our vignettes described legal, well-regulated hunts, so only captured a small segment of the types of activities that are described as ‘trophy hunting' [9]. We expect that perceptions of acceptability would vary more broadly if participants responded to vignettes describing a wider range of activities described as trophy hunting. For example, we would expect ‘canned hunting', in which captive-bred animals are shot within enclosures, or hunts in which revenues are not fairly or transparently distributed, to be perceived as especially unacceptable [9,28,30,80]. On the other hand, we would expect legally trophy hunting certain wild animals, such as reptiles, waterfowl and fishes, to be perceived as more acceptable.

Participants' social identities and orientations towards people and wild animals can be stronger predictors of perceptions of the acceptability of trophy hunting than specific attributes of a hunt (figure 3). For some participants, such as those who strongly identify as hunters or animal protectionists, social identity might be more relevant to how they perceive trophy hunting than attributes of hunts. In our sample, most respondents from all three countries identified to some degree as an animal protectionists but not as a hunter (electronic supplementary material, S2). Our estimates of how these identities predict perceptions of the acceptability of trophy hunting, particularly towards the strongly agree end of the hunter scale and the strongly disagree end of the animal protectionist scale, therefore include substantial uncertainty (electronic supplementary material, S6; figure 3). Nevertheless, our findings echo other studies that have documented associations between individuals' social identities and their positions on controversial issues in wildlife conservation [81,82] and imply that disagreements about the acceptability of trophy hunting well-known African animals may, at least in part, be a form of identity politics. In our study, the social identities in question are being a hunter or an animal protectionist but not the alternatives we also evaluated, namely being an advocate for conservation or being an advocate for human rights, neither of which appeared in our top-supported model. However, beliefs about whether to prioritize the interests of people or wild animals when their interests clash did appear in our top-supported model, suggesting that long-standing tensions within the conservation community about conflicts between human and non-human interests [77,83] are also relevant to how members of the public evaluate contentious issues in conservation.

We expected to find evidence for associations between additional demographic characteristics and perceptions of the acceptability of trophy hunting (electronic supplementary material, S1). In particular, we predicted that men would perceive trophy hunting as more acceptable than women, and older people would perceive it as more acceptable than younger people [84] (electronic supplementary material, S1). However, the only demographic characteristics that appeared in our top-supported model were participants' country of residence and their level of formal education, which had a positive but weak association with perceptions of the acceptability of trophy hunting (figure 3). One possible explanation for this finding is that participants with more formal education may have been more familiar with the ecological, social, and economic complexities of trophy hunting. However, we expect that people will be more likely to become familiar with those complexities if they have direct experience of hunting or experience of life in rural Africa, regardless of how much formal education they have completed. For example, conservationists who are from or who have worked in Africa tend to adopt a more people-centred view on conservation, and to express more favourable views towards trophy hunting [6,85]. However, we found no evidence that perceptions of the acceptability of trophy hunting differed between participants who grew up in a rural location, and might therefore have had more exposure to hunting, compared to those who grew up in an urban location. It is possible that there is a link between level of formal education, income, and the ability to travel, with people who have travelled more widely gaining a better understanding of conditions and needs on the African continent.

## 5. Conclusion

It is important to understand how members of external publics think about trophy hunting because their perceptions and preferences can influence decisions, policies, programmes and funding flows in the international system of biodiversity conservation [6]. We contribute evidence from a study involving members of three publics external to rural sub-Saharan Africa, demonstrating that perceptions of the acceptability of trophy hunting well-known African animals depend on specific attributes of a hunt as well as participants' characteristics.

However, it will be especially important also to investigate perspectives among people who live in rural areas of sub-Saharan Africa. People living in many rural areas of the Global South have historically been marginalized by international conservation and tend to have been overlooked in decisions affecting wildlife where they live [40], even though they are most affected by those decisions [35,86]. Research that focuses only on external perspectives risks perpetuating inequalities in international conservation [13,35], but research that encompasses and integrates external and local perspectives could provide evidence to inform difficult and contentious decisions about the role of trophy hunting in wildlife conservation and economic development [14].

## Data Availability

Data available from the Dryad Digital Repository: (https://doi.org/10.5061/dryad.bvq83bkfr) [74]. Extra information is provided in the electronic supplementary material [87].
